# Editorial: γδ T Cells in Cancer

**DOI:** 10.3389/fimmu.2020.602411

**Published:** 2020-11-20

**Authors:** Seth B. Coffelt, Dieter Kabelitz, Bruno Silva-Santos, Jurgen Kuball, Willi Born, Ilan Bank

**Affiliations:** ^1^ Institute of Cancer Sciences, University of Glasgow, Glasgow, United Kingdom; ^2^ Cancer Research UK Beatson Institute, Glasgow, United Kingdom; ^3^ Institute of Immunology, University of Kiel, Kiel, Germany; ^4^ Faculdade de Medicina, Instituto de Medicina Molecular, Universidade de Lisboa, Lisbon, Portugal; ^5^ Department of Hematology and Center for Translational Immunology, Utrecht Medical Center (UMC), Utrecht, Netherlands; ^6^ Department of Immunology and Genomic Medicine, National Jewish Health, Denver, CO, United States; ^7^ Rheumatology Unit, Division of Medicine, Sheba Medical Center, Tel Hashomer, Israel

**Keywords:** γδ T cells, cancer, butyrophilin, immunotherapy, bisphoshonates

Since the discovery of γδ T cells, this rare and unique component of the immune system has been recognized for its potential in cancer immunology and immunotherapy. In the mid-1980s, it became clear that a major component of adaptive immune responses is the ability of T cell receptors (TCR) to undergo somatic recombination in order to recognize multiple antigens. TCRs consisting of either αβ and γδ chains were discovered in rapid succession (1–6). An important observation was made in these initial studies: γδ T cells stimulated through their TCR are able to kill cancer cells (2). Over these past decades, researchers have learned that γδ T cells share many similarities with αβ T cells, as well as major differences. However, discoveries in γδ T cell biology have failed to keep the same pace as αβ T cell biology. The molecular targets of γδTCRs and functions of these cells have largely eluded researchers, partly because γδ T cell recognition of cancer cells and their response kinetics are very different to αβ T cells (7, 8). Recent years have seen major advances in γδ T cell biology and established the non-redundancy of this lymphocyte subset, particularly in the context of cancer (9–11). γδ T cells are being used as cellular vehicles to target tumors and prognostic indicators of cancer progression. The aim of the articles collected in this Research Topic is to describe new developments and approaches to enhance the anti-tumor functions of γδ T cells, and to discuss how expression of their ligands can assist with prognosis of cancer patients.

Given the robust ability of γδ T cells to kill cancer cells, various strategies to enhance their cytotoxic behavior are being pursued in the laboratory. The Vγ9Vδ2 cell subset in humans recognizes transformed cells with dysfunctional metabolism, primarily through the up-regulation of phosphoantigens stemming from abnormalities in the mevalonate pathway. One of the best studied phosphoantigens is isopentenyl pyrophosphate (IPP), which activates a receptor complex in cancer cells consisting of butyrophilin (BTN)-3A1 and BTN2A1 (12, 13). However, very little information exists on how this receptor complex is presented on the cell surface and its other interacting partners. Laplagne et al. report on the importance of the GTPase, RhoB, in regulating BTN3A1 presentation on the cell membrane. They observed that the differential susceptibility of lung tumor cell lines to Vγ9Vδ2 T-cell killing correlated with differential subcellular and plasma membrane distribution of RhoB. There are a few methods to increase Vγ9Vδ2 cell recognition of cancer cells, primarily *via* boosting the IPP-activated BTN3A1/BTN2A1 complex. Bisphosphonate drugs increase accumulation of IPP making cancer cells more susceptible to Vγ9Vδ2 cell killing, but these drugs also induce proliferation of Vγ9Vδ2 cells in culture. Okuno et al. report on a newly synthesized bisphosphonate drug, pivaloyloxymethyl 2-(thiazole-2-ylamino)ethylidene-1,1-bisphosphonate (PTA), that both expands Vγ9Vδ2 cells and increases their ability to recognize cancer cells.

γδ T cells are also being equipped with chimeric antigen receptors (CAR) for hematological and epithelial-derived malignancies. Most likely, γδ CAR T cells will associate with a lower risk of cytokine release syndrome as reported for NK CAR cells. Whether γδ CAR T cells will overcome the scarce infiltration of tumors by classical αβ CAR T cells remains to tested and might depend on the type of γδ T cells (i.e. Vδ1 cells versus Vδ2 cells), which have naturally different homing tissues. Rozenbaum et al. describe a new expansion protocol that generates high numbers of pure (> 99%) γδ T cells which could be efficiently transduced with CAR constructs. CD19-directed γδ CAR T cells efficiently killed CD19+ leukemic cells *in vitro* and *in vivo*. To test these various strategies whose goal is augment γδ T cell cytotoxic function, a variety of pre-clinical models are used that evaluate killing efficacy, but these models come with their own challenges. Joalland and Scotet summarize the advantages and disadvantages of the most commonly used pre-clinical models in γδ T cell immunotherapy. In addition to the use of immunodeficient mice transplanted with human tumor cells and γδ T cells, they also discuss the urgent need for improved animal-free *in vitro* models such as spheroids and organoids.

Another outstanding question in the field is how γδ T cell function may be suppressed by tumors. Siegers et al. show that an embryonic-associated molecule, called NODAL, expressed by breast cancer cells, impacts γδ T cell function. In human breast tumors, γδ T cells are found in close proximity to NODAL+ cancer cells. Through gain-of-function and loss-of-function experiments, the authors report that NODAL expression on breast cancer cell lines reduces γδ T-cell cytotoxicity. Gonnermann et al. describe a novel immunosuppression pathway in pancreatic cancer, where Galectin-3 secreted by cancer cells inhibits γδ T cell proliferation *via* α3β1 integrin. Interestingly, the cytotoxic activity of γδ T cells was not impaired by Galectin-3.

This Research Topic includes two clinical trials. One trial investigated the ability of β-adrenergic receptor activation to mobilize γδ T cells into the peripheral blood of test subjects. Baker et al. found that drugs antagonizing the β-adrenergic receptor pathway not only prevent γδ T cell accumulation in blood, but that this pathway is also important for γδ T cell expansion *ex vivo*. These data suggest that β-adrenergic receptor agonists may improve expansion protocols or γδ T cell cytotoxic function. The second clinical trial included 46 children with acute leukemia that received hematopoietic stem cell transplantation of αβTCR/CD19-depleted haploidentical grafts. Merli et al. tested the ability of the bisphosphonate drug, zoledronic acid, to counteract graft-versus-host-disease in these patients, and they show that zoledronic acid is well tolerated. Moreover, the children that received more doses of zoledronic acid had a better outcome than the children receiving fewer doses.

Finally, two articles in this collection discuss the importance of γδ T cell ligands in cancer immunotherapy. Bartish et al. summarize the role of immunosuppressive molecules that contain sugar residues as well as the relationship between these glyco-molecules and γδ T cells, highlighting opportunities for intervention. Wang et al. wrote a meta-analysis on publications pertaining to the BTN family. In this review, the authors also provide prognostic data for several BTN family members in lung adenocarcinoma and lung squamous cell carcinoma.

Together, this Research Topic features new developments in γδ T cell cancer immunotherapy, providing insight into mechanisms that both increase and suppress their effector functions. Finding the right patient population in which to manipulate these pathways and exploit this new information may be key to counteracting cancer.

## Author Contributions

SC, DK, BS-S, JK, WB, and IB wrote the editorial and invited authors to participate in the collection. All authors contributed to the article and approved the submitted version.

## Funding

The authors acknowledge funding from the Cancer Research UK Glasgow Centre (A25142 to SBC), the Wellcome Trust (208990/Z/17/Z to SBC), the Medical Research Council (MR/R502327/1 to SBC), Breast Cancer Now (2018JulPR1101 and 2019DecPhD1349 to SBC), Tenovus Scotland (S17-17 to SBC), the Deutsche Forschungsgemeinschaft (Ka 502/19-2 to DK), the Wilhelm Sander Foundation (2018.045.1 to DK), “la Caixa” Banking Foundation (HR18-00069 to BS-S), the Dutch Research Council (NWO ZonMW 43400003 to JK), the Dutch Cancer Society (KWF UU 2014-6790, UU 2015-7601, UU 2018-11393, UU 2018-11979, UU 2019-12586, UU 2020-13043 to JK).

## Conflict of Interest

BS-S is a founder and share holder of Lymphact S.A., which has been acquired by GammaDelta Therapeutics (London, UK). JK is scientific co-founder and shareholder of Gadeta. JK received financial research support of Gadeta, Miltenyi Biotech and Novartis.

The remaining authors declare that the research was conducted in the absence of any commercial or financial relationships that could be construed as a potential conflict of interest.
